# George E. Hawes

**Published:** 1875-03

**Authors:** 


George E. Hawes, D. D. S., who died in December, 1874, also received the degree of D. D. S., in 1847, from the Baltimore College of Dental Surgery. Dr. Hawes was a native of Wrentham, Mass., and successfully practiced his profession in New York city, from the commencement of his professional career, up to the time of his death. Besides being eminent in his profession, he was appreciated for all those qualities which adorn and honor the possessor in every walk in life. His name has also become widely known in connection with Hawes Tongue and Duct Compressor, almost universally used prior to the introduction of the Rubber Dam.

